# Galectin-1 Impairs the Generation of Anti-Parasitic Th1 Cell Responses in the Liver during Experimental Visceral Leishmaniasis

**DOI:** 10.3389/fimmu.2017.01307

**Published:** 2017-10-12

**Authors:** Patrick T. Bunn, Marcela Montes de Oca, Fabian de Labastida Rivera, Rajiv Kumar, Chelsea L. Edwards, Rebecca J. Faleiro, Susanna S. Ng, Meru Sheel, Yulin Wang, Fiona H. Amante, Ashraful Haque, Christian R. Engwerda

**Affiliations:** ^1^QIMR Berghofer Medical Research Institute, Brisbane, QLD, Australia; ^2^Institute of Glycomics, Griffith University, Gold Coast, QLD, Australia; ^3^Department of Biochemistry, Banaras Hindu University, Varanasi, India; ^4^School of Medicine, University of Queensland, Brisbane, QLD, Australia; ^5^School of Natural Sciences, Griffith University, Nathan, QLD, Australia; ^6^School of Chemistry and Molecular Biosciences, University of Queensland, Brisbane, QLD, Australia

**Keywords:** *Leishmania*, visceral leishmaniasis, galectin-1, T cells, inflammation

## Abstract

Many infectious diseases are characterized by the development of immunoregulatory pathways that contribute to pathogen persistence and associated disease symptoms. In diseases caused by intracellular parasites, such as visceral leishmaniasis (VL), various immune modulators have the capacity to negatively impact protective CD4^+^ T cell functions. Galectin-1 is widely expressed on immune cells and has previously been shown to suppress inflammatory responses and promote the development of CD4^+^ T cells with immunoregulatory characteristics. Here, we investigated the role of galectin-1 in experimental VL caused by infection of C57BL/6 mice with *Leishmania donovani*. Mice lacking galectin-1 expression exhibited enhanced tissue-specific control of parasite growth in the liver, associated with an augmented Th1 cell response. However, unlike reports in other experimental models, we found little role for galectin-1 in the generation of IL-10-producing Th1 (Tr1) cells, and instead report that galectin-1 suppressed hepatic Th1 cell development. Furthermore, we found relatively early effects of galectin-1 deficiency on parasite growth, suggesting involvement of innate immune cells. However, experiments investigating the impact of galectin-1 deficiency on dendritic cells indicated that they were not responsible for the phenotypes observed in galectin-1-deficient mice. Instead, studies examining galectin-1 expression by CD4^+^ T cells supported a T cell intrinsic role for galectin-1 in the suppression of hepatic Th1 cell development during experimental VL. Together, our findings provide new information on the roles of galectin-1 during parasitic infection and indicate an important role for this molecule in tissue-specific Th1 cell development, but not CD4^+^ T cell IL-10 production.

## Introduction

Dysregulated cellular immune responses are a feature of many chronic infectious diseases (1). Visceral leishmaniasis (VL) caused by the protozoan parasites *Leishmania donovani* and *L.infantum* (*chagasi*) represents one such disease. VL is present in the Indian sub-continent, the Americas, Mediterranean Basin, and East Africa. There are between 200,000 and 400,000 VL cases and around 30,000 deaths each year (2). In VL patients, *Leishmania* parasites rapidly infect macrophages throughout the viscera and become established in the liver, spleen, lymph nodes, and bone marrow (BM) (3). To date, much of our knowledge regarding host immune responses to *L. donovani* comes from studies in genetically susceptible C57BL/6 and BALB/c mice, which display tissue-specific immune responses to infection. The liver is a site of acute, resolving infection, while chronic infections are established in the spleen and BM (3, 4). These disparate responses appear to reflect aspects of asymptomatic infection and fulminant disease in humans, respectively (3, 4). While the immune mechanisms involved in hepatic parasite control have been extensively characterized, the underlying causes of parasite persistence in the spleen and BM are less well understood. The establishment of protective immunity is critically dependent on the generation of pro-inflammatory CD4^+^ T cells producing IFNγ and TNF (5, 6). These Th1 cells subsequently promote antimicrobial activity in parasitized macrophages (7). However, chronic disease is characterized by the establishment of potent immunoregulatory networks causing profound impairment in these protective immune responses (4).

A better understanding of immunoregulatory networks will be crucial for future efforts to treat chronic infection. One of the most potent immunoregulatory molecules identified to date in both mouse models of VL and VL patients is IL-10. While IL-10 signaling appears to be necessary for restricting tissue damage that occurs as a result of excessive inflammation (8), both experimental (9, 10) and clinical (11–15) data suggest that this immunoregulatory cytokine contributes to the establishment and/or maintenance of chronic infection during VL. Similar roles for IL-10 have also been described in other infectious diseases, including tuberculosis (16), toxoplasmosis (17), and malaria (18). In C57BL/6 mice infected with *Plasmodium chabaudi* AS, IL-10 deficiency had a minimal impact on parasite growth but caused significant pathology, as indicated by increased anemia and liver damage (19).

Galectin-1 is the prototypical member of a large family of β-galactoside-binding proteins, collectively known as galectins, involved in a wide range of immunomodulatory functions (20). Indeed, all immune cells express galectins to varying extents, though they are notably upregulated on activated B cells, NK cells, macrophages, and both conventional T cells and FoxP3^+^ regulatory T (Treg) cells (21). The pleiotropic nature of galectin-1 arises, in part, on the distribution of the functionally disparate intracellular and extracellular forms of the molecule on different cell populations (20). Intracellular galectin-1 exists primarily in monomeric form and regulates cell growth *via* interactions with Ras family proteins (22). Conversely, the dimeric form of galectin-1 is responsible for lectin activity, which acts as a negative regulator of immune responses (23). Upon secretion, galectin-1 spontaneously dimerizes, whereupon the stability and functionality of the protein is critically dependent on rapid binding to extracellular glycan ligands (23, 24). Previously described functions for galectin-1 in the context of effector T cell regulation include the induction of apoptosis in effector lymphocytes (25–27) and the promotion of immunoregulatory T cell phenotypes (28–30). In addition, Foxp3^+^ Treg cell suppressive dysfunction has been reported in galectin-1-deficient (*Lgals1^−/−^*) mice (31), suggesting that galectin-1 is required for optimal Treg cell function. *Lgals1^−/−^* mice also exhibit increased pro-inflammatory cytokine production (32), and are more susceptible to autoimmune disease than their wild-type (WT) counterparts (31). Recombinant galectin-1 has been tested as a therapeutic agent in various models of inflammatory disease including arthritis (33), hepatitis (34), type-1 diabetes (35), and graft-versus-host disease (36). Conversely, galectin-1 has been implicated in the promotion of cancer cell immune evasion (37, 38), and blockade of tumor-derived galectin-1 promotes tumor rejection *via* the augmentation of pro-inflammatory T cell responses (39). Similarly, galectin-1 exacerbates disease in models of Hodgkin’s lymphoma by inducing Th2 polarization and expansion of Treg cell populations that impair antitumor responses (40). Neutralizing antibodies (41) and effective inhibitors of galectin-1 binding (42) are currently being evaluated as therapeutic agents in clinical trials aimed at treating various cancers.

One of the important consequences of galectin-1 interactions with T cells is the polarization of naïve and effector T cells to a regulatory phenotype. Naïve T cells stimulated with recombinant galectin-1 *in vitro* rapidly differentiate into an IL-10-producing Th1 (Tr1) cell phenotype (28). This process occurs in either the presence or absence of APC (29), suggesting that galectin-1 can act directly or indirectly on T cells to alter their function. Galectin-1 can additionally enhance the production of IL-10 by Tr1 cells *via* the generation of tolerogenic dendritic cells (DCs) by ligation to CD43 on the DC surface and the subsequent promotion of IL-27 secretion (30), which stimulates IL-10 production by Tr1 cells. This mechanism of galectin-1 immunoregulatory function was shown to contribute to enhanced parasite control, survival, and Th1 effector function in *Lgals1^−/−^* mice infected with *Trypanosoma cruzi* (43). However, in this latter study, galectin-1 promoted DC-mediated induction of Treg cells rather than Tr1 cells.

To determine whether similar mechanisms of galectin-1-mediated immune regulation influenced disease outcome in another important parasitic disease, we infected *Lgals1^−/−^* mice and WT controls with *L. donovani* and assessed control of parasite growth and associated immune responses. Here, we show that galectin-1 suppressed control of hepatic parasite growth without modulating the induction of Tr1 cells. Instead, galectin-1 restricted optimal Th1 cell effector function in the liver. These findings extend our understanding of the diverse roles for galectin-1 in infectious diseases, as well as providing insight into how modulation of galectin-1 may be harnessed for therapeutic advantage.

## Materials and Methods

### Infections

*Leishmania donovani* (LV9; MHOM/ET/67/HU3) was originally isolated from a patient in Ethiopia in 1967 and subsequently maintained by animal passage (44). The parasite line was transferred from the London School of Hygiene and Tropical Medicine (London, UK) to QIMR Berghofer in 2002 and maintained by passage in B6.Rag1^−/−^ mice. Amastigotes were isolated from chronically infected passage animals. Experimental mice were infected by injection of 2 × 10^7^ amastigotes i.v. *via* the lateral tail vein. Cohorts were culled at respective time-points post-infection (p.i.) by CO_2_ asphyxiation and bled *via* cardiac puncture. Spleens were removed and livers perfused then removed, with parasite burden determined *via* histological assessment of Giemsa-stained (Diff-Quick; Lab Aids) liver and spleen tissue impressions and expressed in Leishman–Donovan units; calculated as the number of parasites per 1,000 nuclei multiplied by the organ weight. Hepatic, splenic, and BM mononuclear populations were isolated as previously described (10, 45).

### Mice

Inbred female C57BL/6 and congenic B6.CD45.1 mice, 6 weeks of age, were purchased from the Animal Resource Centre (Canning Vale, WA, Australia). C57BL/6 mice with a specific deletion of the gene-encoding galectin-1 (43) (*Lgals1^−/−^*) were obtained from the Jackson Laboratory and, along with B6.CD11c.DOG mice (46), were bred in-house at QIMR Berghofer (Brisbane, QLD, Australia). All mice were age- and sex-matched and maintained in-house under pathogen-free conditions. All animal procedures were conducted with the approval of the QIMR Animal Ethics Committee under the animal ethics number A02-634M and in accordance with the “Australian Code of Practice for the Care and Use of Animals for Scientific Purposes” (Australian NHMRC, Canberra).

### Generation of Mixed BM Chimeric Mice

Chimeric mice were generated by lethally irradiating mice with two doses of 5.5 cGy and subsequently engrafting with 10^6^ freshly isolated BM cells i.v. *via* the lateral tail vein, as previously described (47). To examine the consequences of antigen presentation by DC in the absence of galectin-1 signaling, a 50:50 mix of B6.CD11c.DOG and either *Lgals1^−/−^* or congenic WT B6.CD45.1 BM cells were engrafted into a WT recipient. Mice were maintained on pyrimethamine/neomycin sulfate for 2 weeks post-engraftment and infected approximately 8–12 weeks thereafter, as previously described (48). Following cellular reconstitution and subsequent infection, mice harboring a B6.CD11c.DOG hematopoietic compartment received 8 ng/g body weight diphtheria toxin (DT) intraperitoneally (i.p.) every 3 days over a 2-week period, whereupon animals were euthanized. This strategy resulted in all B6.CD11c.DOG DC being depleted by DT, leaving behind only WT or *Lgals1^−/−^* DC for antigen presentation to T cells.

A second set of mixed BM chimeras were generated as above, except that irradiated recipients were engrafted with a 90:10 mix of congenic (CD45.1) WT (90%) and either *Lgals1^−/−^* or WT (both CD45.2) (10%) BM cells. This strategy allowed *Lgals1^−/−^* CD4^+^ T cells activity to be measured in a predominantly WT immunological background and compared with appropriate WT control CD4^+^ T cells following infection.

### Flow Cytometry

All organ-derived mononuclear cells were prepared as described previously (10, 47, 49). Fluorescently conjugated mAbs against CD4 (GK1.5), CD8α (53-6.7), TCRβ (H57-597), B220 (RA3-6B2), CD19 (6D5), Foxp3 (MF-14), IFNγ (XMG1.2), IL-10 (JES5-16E3), TNF (MP6-XT22), CD11c (N418), CD11b (M1/70), MHC-II (M5/114.15.2), F4/80 (BM8), Ly6C (HK1.4), Ly6G (IA8), NK1.1 (PK136), CD45.1 (A20), CD45.2 (104) (Biolegend, San Diego, CA, USA), and Galectin-1 (R&D Systems, Minneapolis, MN, USA) were used. Dead cells were excluded from analysis using LIVE/DEAD Fixable Aqua Stain (Invitrogen), as per the manufacturer’s instruction. Both cell surface and intracellular staining was undertaken according to methods described previously (10), with all samples acquired on a BD LSRFortessa (BD Biosciences). Gating strategies used for analysis are outlined in the manuscript. For analysis of intracellular IL-10, cells were stimulated for 3 h at 37°C and 5% CO_2_ in the presence of PMA and ionomycin in addition to Brefeldin A, as described previously (10).

### Statistical Analysis

Statistical analysis was performed exclusively in GraphPad Prism 5 and 6 (GraphPad Software, La Jolla, CA, USA). A non-parametric, unpaired Mann–Whitney test was used for comparisons between two groups. A *p* value of <0.05 was considered significant. Graphs depict mean ± SEM.

## Results

### Galectin-1 Is Upregulated on T Cell Subsets following Establishment of *L. donovani* Infection

**Figure 1:**
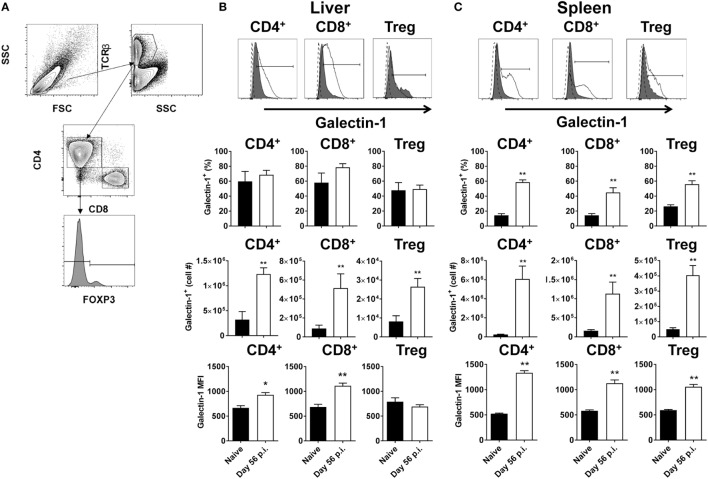
Galectin-1 expression on T cell subsets during established infection. Galectin-1 expression on T cell subsets from C57BL/6 mice (*n* = 5) infected with *Leishmania donovani* (day 56 p.i.) was assessed by flow cytometry and compared with naïve control animals. The gating strategy used to identify conventional (Foxp3^−^) CD4^+^, CD8^+^, and regulatory (Foxp3^+^) T cells is shown **(A)**. The frequency and number of galectin-1-positive cells, as well as the relative expression of galectin-1 determined by mean fluorescent intensity (MFI) from histograms shown for the liver **(B)** and spleen **(C)** were measured, as indicated. The dotted histograms represent staining with the isotype control antibody, dark gray histograms reflect cells from naïve animals, and white histograms reflect cells from infected animals. These data represent one of two separate experiments (*p < 0.05, **p < 0.01; Mann–Whitney test, relative to naïve).

Antigen-presenting cells expressing galectin-1 have previously been shown to promote an IL-10-mediated immunoregulatory pathway (30). To determine whether T cells expressing galectin-1 might also play immunoregulatory roles, T cell subsets from livers and spleens of *L. donovani*-infected C57BL/6 mice at day 56 p.i. were assessed for galectin-1 expression (Figure 1A). We chose this time point because infection had largely resolved in the liver and effective CD4^+^ T cell-mediated, concomitant immunity was established. In contrast, the spleen was a site of chronic infection associated with dysregulated CD4^+^ T cell responses (50, 51). Thus, day 56 p.i. represents two extremes of infection outcome. Elevated numbers of galectin-1-expressing CD8^+^ and CD4^+^ T cells, including Foxp3^+^ Treg cells, were observed in both liver (Figure 1B) and spleen (Figure 1C), although increased frequencies of galectin-1-expressing T cell subsets was only found in the latter tissue. However, the level of expression on a per-cell basis [i.e., the galectin-1 mean fluorescent intensity (MFI) value] was significantly (*p* < 0.05 and *p* < 0.01) higher than naïve equivalents for all populations, except hepatic Foxp3^+^ Treg cells (Figures 1B,C).

**Figure 2:**
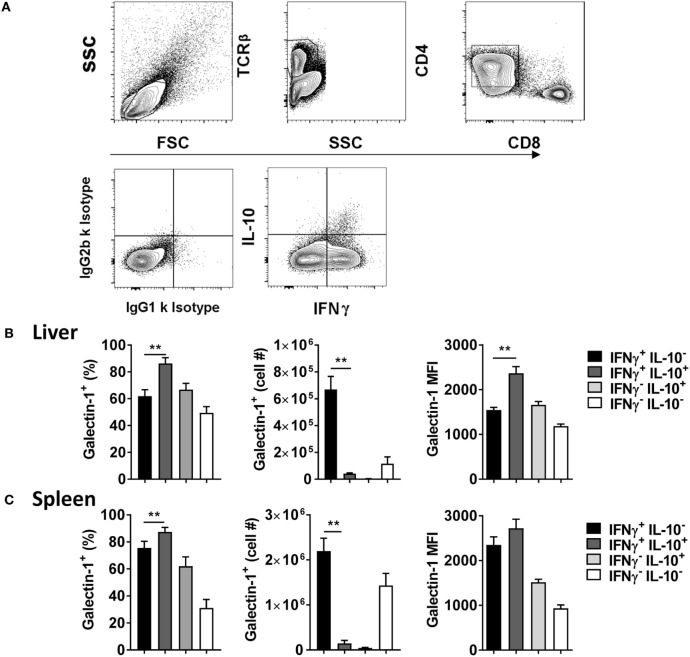
Galectin-1 expression on IFNγ- and IL-10-producing CD4^+^ T cells during established infection. IFNγ and IL-10 production by CD4^+^ T cells from C57BL/6 mice (*n* = 5) infected with *Leishmania donovani* (day 56 p.i.) was assessed by flow cytometry. The gating strategy used to identify cytokine-producing CD4^+^ T cells is shown **(A)**. The frequency and number of galectin-1-positive cytokine-producing cells, as well as the relative expression of galectin-1 determined by mean fluorescent intensity (MFI), on these cells in the liver **(B)** and spleen **(C)** were measured, as indicated. These data represent one of two separate experiments (**p < 0.01; Mann–Whitney test between IL-10^−^ IFNγ^+^ and IL-10^+^ IFNγ^+^ CD4^+^ T cells only is shown).

Given previous association between galectin-1 and IL-10-producing T cells (28–30), and the importance of Tr1 cells for disease outcome in experimental VL (10), the level of galectin-1 expression on IL-10 and IFNγ-producing CD4^+^ effector cells was next examined (Figure 2A). We found that a greater frequency of IL-10^+^ IFNγ^+^ Tr1 cells expressed galectin-1, compared with IL-10^−^ IFNγ^+^ Th1 cells, in the liver (Figure 2B) and spleen (Figure 2C), but expression was only significantly (*p* < 0.01) higher on a per-cell basis in the liver. However, given the lower number of Tr1 cells, relative to Th1 cells (Figure 2A), the number of Tr1 cells expressing galectin-1 was significantly (*p* < 0.01) lower than Th1 cells in both organs studied (Figures 2B,C). Thus, galectin-1 was highly expressed by Tr1 cells, but the number of Th1 cells expressing this molecule in both tissues was significantly greater at the day 56 p.i. time point examined.

### Tissue-Specific Upregulation of Galectin-1 on Non-T Cell Populations

**Figure 3:**
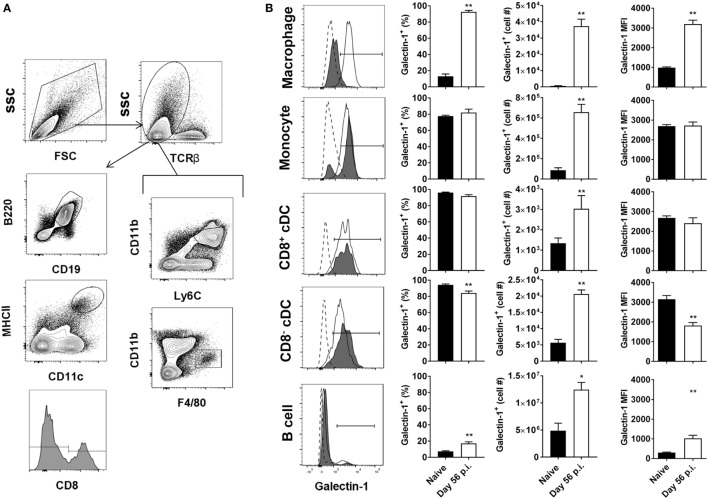
Galectin-1 expression on hepatic antigen-presenting cells during established infection. Galectin-1 expression on antigen-presenting cells from C57BL/6 mice (*n* = 5) infected with *Leishmania donovani* (day 56 p.i.) was assessed by flow cytometry and compared with naïve control animals. The gating strategy used to identify antigen-presenting cells in the liver is shown **(A)**. The frequency and number of galectin-1-positive cells, as well as the relative expression of galectin-1 determined by mean fluorescent intensity (MFI) from histograms, were measured, as indicated **(B)**. Dotted histograms reflect isotype control antibody staining, dark gray histograms reflect cells from naïve animals, and white histograms reflect cells from infected animals. These data represent one of two separate experiments (**p < 0.01; Mann–Whitney test, relative to naïve).

**Figure 4:**
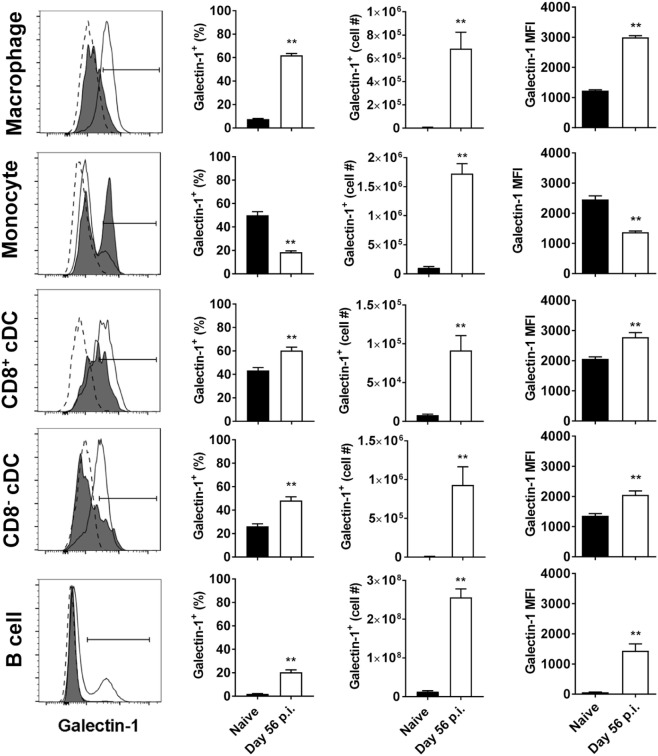
Galectin-1 expression on splenic antigen-presenting cells during established infection. Galectin-1 expression on antigen-presenting cells from C57BL/6 mice (*n* = 5) infected with *Leishmania donovani* (day 56 p.i.) was assessed by flow cytometry and compared with naïve control animals. The gating strategy used to identify antigen-presenting cells in the spleen was the same as shown in Figure 3A. The frequency and number of galectin-1-positive cells, as well as the relative expression of galectin-1 determined by mean fluorescent intensity (MFI) from histograms, were measured, as indicated. Dotted histograms reflect isotype control antibody staining, dark gray histograms reflect cells from naïve animals, and white histograms reflect cells from infected animals. These data represent one of two separate experiments (**p < 0.01; Mann–Whitney test, relative to naïve).

We next investigated galectin-1 expression patterns on antigen-presenting cell populations at the same time point (i.e., day 56 p.i.). Galectin-1 expression (as determined by MFI) was substantially upregulated on hepatic (Figure 3) and splenic (Figure 4) macrophages, compared to naïve counterparts. Galectin-1 expression on monocytes did not change in the liver (Figure 3) and decreased in the spleen (Figure 4), but due to increased leukocyte numbers in these tissues as a result of infection (47), the number of monocytes expressing galectin-1 was increased in both tissues (Figures 3B and 4). Hence, in the spleen, there were more monocytes expressing lower levels of galectin-1 in infected mice, compared to naïve counterparts. Other tissue-specific discrepancies in galectin-1 expression were observed on DC subsets, with increased expression noted on splenic CD8^+^ and CD8^−^ DC, but not on the same DC subsets in the liver (Figures 3B and 4). A small subpopulation of B cells was found to express galetin-1 during established infection in both the liver and spleen, although this population and galectin-1 expression was more prominent in the spleen (Figures 3B and 4). Therefore, galectin-1 was expressed by multiple antigen-presenting cell populations during established *L. donovani* infection, but expression varied, depending on cell type and tissue location.

### The Impact of Galectin-1-Deficiency on *L. donovani* Infection

**Figure 5:**
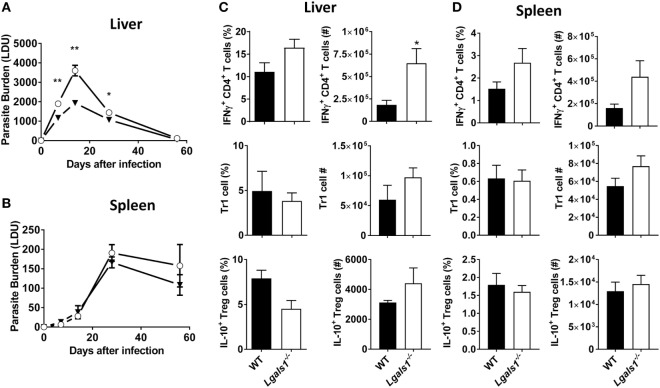
Impact of galectin-1-deficiency on control of *Leishmania donovani*. Parasite burdens in C57BL/6 [wild-type (WT); open circles] and *Lgals1^−/−^* (closed triangles) mice (*n* = 4–5 per time point) infected with *L. donovani* were measured in the liver **(A)** and spleen **(B)**, as indicated, on days 7, 14, 28, and 56 p.i. The frequencies and total numbers of IFNγ-producing CD4^+^ T cells, Tr1 cells, and IL-10-producing Treg cells in the liver **(C)** and spleen **(D)** at day 14 p.i. were measured, as indicated. These data represent one of three separate experiments (*p < 0.05, **p < 0.01; Mann–Whitney test, *Lgals1^−/−^* relative to WT at time-point assessed).

Given the increased expression of galectin-1 on key immune cell populations in infected tissues, the influence of galectin-1 on *L. donovani* infection was assessed using mice lacking galectin-1 expression (*Lgals1^−/−^*). *Lgals1^−/−^* mice exhibited significantly (*p* < 0.05 and *p* < 0.01) reduced parasite burdens in the liver during the acute phase of infection, as compared to WT control animals (Figure 5A). However, splenic parasite burdens remained unchanged between *Lgals1^−/−^* mice and controls across the course of infection (Figure 5B). Galectin-1 has previously been associated with increased IL-10 production in conventional and regulatory CD4^+^ T cell subsets (30). In addition, we recently reported that IL-10 production by Tr1 cells suppressed anti-parasitic immunity in the liver of *L. donovani*-infected mice (10). Therefore, subsequent cellular analysis focused on alterations in pro- and anti-inflammatory responses by key CD4^+^ T cell subsets in the liver and spleen. An increased frequency of IFNγ-producing CD4^+^ T cells was found in the livers of *Lgals1^−/−^* mice at day 14 p.i., which translated to significant (*p* < 0.05) increase in the total number of IFNγ-producing cells in this organ, compared with control animals (Figure 5C). Despite a similar trend in the spleen, differences in frequency and number of IFNγ-producing cells were not significantly different (Figure 5C). Both regulatory (Foxp3^+^) and conventional (Foxp3^−^) CD4^+^ T cells have the potential to produce IL-10 during *L. donovani* infection (10). However, no difference in the frequency or number of either of these IL-10-producing CD4^+^ T cells was observed in the liver (Figure 5C) or spleen (Figure 5D) at days 14 p.i. Together, these data indicate that galectin-1 modulates protective CD4^+^ T cell responses during acute, hepatic *L. donovani* infection by limiting numbers of IFNγ-producing CD4^+^ T cell numbers, and not by promoting CD4^+^ T cell IL-10 production.

### Galectin-1 Expression Early during *L. donovani* Infection

**Figure 6:**
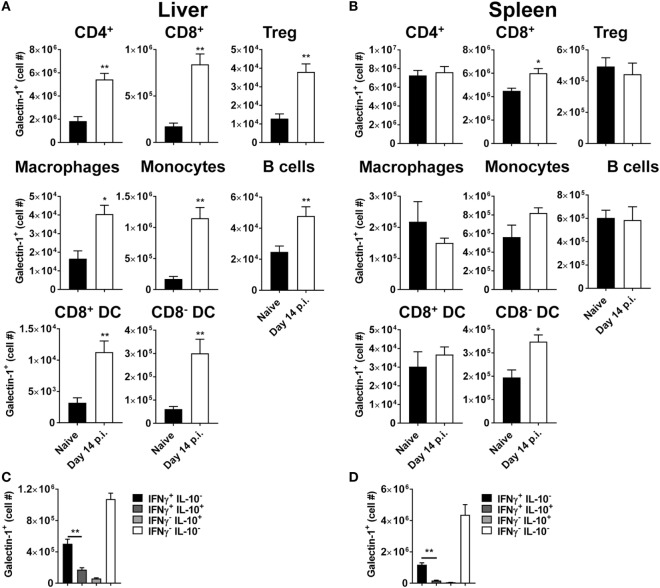
Galectin-1 expression on immune cells early during *Leishmania donovani* infection. Galectin-1 expression on immune from C57BL/6 mice (*n* = 5) infected with *L. donovani* on day 14 p.i. was assessed by flow cytometry and compared with naïve control animals. The gating strategy used to identify cells was the same as shown in Figures 1A and 3A. The number of galectin-1-positive cells in the liver **(A)** and spleen **(B)**, as well as the number of galectin-1-positive cytokine-producing cells in the liver **(C)** and spleen **(D)**, were measured, as indicated. These data represent one of two separate experiments [**p < 0.01; Mann–Whitney test, relative to naïve **(A,B)**; Mann–Whitney test between IL-10^−^ IFNγ^+^ and IL-10^+^ IFNγ^+^ CD4^+^ T cells only is shown **(C,D)**].

Given that enhanced control of parasite growth in the liver was observed early after infection, we next examined galectin-1 expression on immune cell populations at day 14 p.i. (Figure 6). Strikingly, the number of galectin-1-positive cells in the liver was elevated for all immune cell subsets examined (Figure 6A), while only numbers of CD8^+^ T cells and CD8^−^ DCs expressing galectin-1 in the spleen were increased (Figure 6B), relative to cells from naïve mice. Of note, the ratio of galectin-1-positive CD4^+^ T cells to galectin-1-positive Treg cells was higher in the liver, compared to the spleen, in naïve animals (143 ± 23 versus 15 ± 3 for liver and spleen, respectively), and these ratios did not change significantly following *L. donovani* infection. Similar to day 56 p.i., the number of Tr1 cells expressing galectin-1 was significantly lower than Th1 cells in both liver (Figure 6C) and spleen (Figure 6D). It should be noted that the number of galectin-1-positive CD4^+^ T cells lacking both IFNγ and IL-10 expression were the most numerous CD4^+^ T cell subset in both tissues studied at this time point (Figures 6C,D). Therefore, galectin-1 expression increased on a broader range of immune cells in the liver than the spleen, and the biggest change in cell numbers occurred in liver CD4^+^ T cells. Given these findings, and the importance of DCs for priming CD4^+^ T cells in experimental VL (47), we next focused on investigating the role of galectin-1 on hepatic DCs and CD4^+^ T cells.

### Galectin-1 Directly Influences Hepatic Th1 Cell Function during Early *L. donovani* Infection

**Figure 7:**
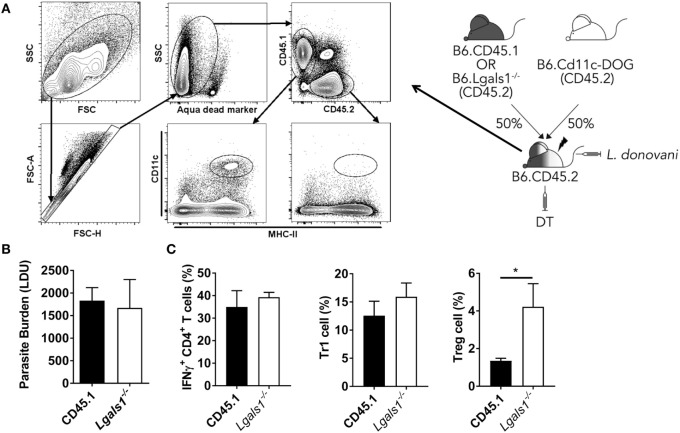
Galectin-1 expression by dendritic cell (DC) has limited impact on infection outcome in the liver. Mixed bone marrow (BM) chimeras including a CD11c. DOG compartment were generated as shown. These mice were infected 3 months after BM engraftment, and received diphtheria toxin (DT) every 2 days from day −1 to day 13 p.i., and DC depletion was confirmed by flow cytometry **(A)**. Liver parasite burdens in DT-treated CD11c.DOG/B6.CD45.1 (CD45.1) and CD11c.DOG/*Lgals1^−/−^* (*Lgals1^−/−^*) chimeras were measured at day 14 p.i., as indicated **(B)**. The frequencies of IFNγ-producing CD4^+^ T cells, Tr1 cells, and Treg cells in the liver were measured at the same time **(C)**. These data represent one of two separate experiments (*n* = 5 mice per group in each; no differences between groups were found, as determined by Mann–Whitney test).

To determine whether galectin-1 mediates its immunoregulatory function through DCs, we generated BM chimeras that comprised a 50:50 mix of CD11c.DOG and WT (CD45.1) or *Lgals1^−/−^* cells. This allowed us to deplete transgenic DCs *via* DT administration, leaving behind only WT or *Lgals1^−/−^* DC for T cell priming, and thus enabling us to determine the impact of DC-expressed galectin-1 on CD4^+^ T cell activation in these animals (Figure 7A). We found no impact on parasite burden in the liver at day 14 p.i., regardless of whether DCs expressed galectin-1 or not (Figure 7B). Furthermore, we found no differences in the frequency of IFNγ-producing CD4^+^ T cells or Tr1 cells in these mice (Figure 7C), indicating that galectin-1 expression by DCs was not responsible for the improved control of parasite growth observed in *Lgals1^−/−^* mice (Figure 5A). Interestingly, we found an increase in the frequency of Treg cells in mice with galectin-1-deficient DCs (Figure 7C).

**Figure 8:**
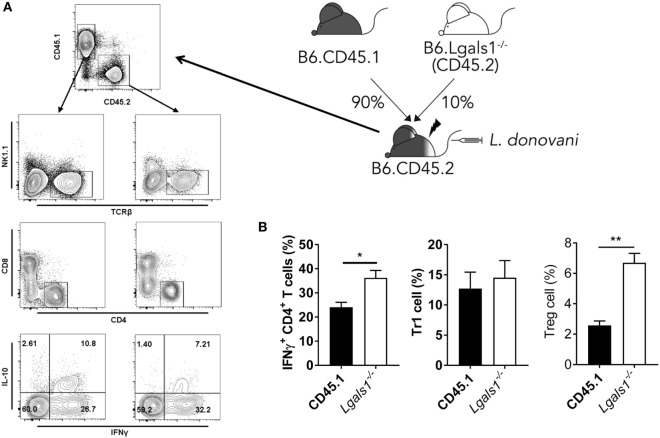
CD4^+^ T cell intrinsic galectin-1-deficiency promotes CD4^+^ T cell IFNγ production in the liver. Mixed bone marrow (BM) CD45.1/*Lgals1^−/−^* (90:10) chimeras were generated as shown. Mice were infected with *Leishmania donovani* 3 months after BM engraftment **(A)**. The frequencies of IFNγ-producing CD4^+^ T cells, Tr1 cells, and Treg cells in the liver were measured at day 14 p.i. **(B)**. Treg cells were gated as described in Figure 1A. These data represent one of two separate experiments [*n* = 5 mice per group in each; **p < 0.01; Mann–Whitney test, wild-type (CD45.1) relative to *Lgals1^−/−^* IFNγ^+^ CD4^+^ T cell frequency at day 14 p.i.].

A potential caveat with the above experiment was that the *Lgals1^−/−^* CD4^+^ T cells were present in the irradiated recipients receiving CD11c.DOG and *Lgals1^−/−^* cells and may have influenced results. To address this, and also test whether there was a T cell intrinsic role for galectin-1 during *L. donovani* infection, we generated another set of BM chimeras that comprised a 90:10 mix of WT (CD45.1) and *Lgals1^−/−^* cells. This allowed *Lgals1^−/−^* CD4^+^ T cell activity to be measured in a predominantly WT immunological background and compared with appropriate WT control CD4^+^ T cells following infection (Figure 8A). We found a small, but significant (*p* < 0.05) difference in the frequency of hepatic IFNγ-producing *Lgals1^−/−^* CD4^+^ T cells, compared to WT CD4^+^ T cells, but no difference in Tr1 cell frequency in the 90:10 chimeras after 14 days of *L. donovani* infection (Figure 8B). However, we found an increased frequency of galectin-1-deficient hepatic Treg cells (Figure 8B), suggesting that similar galectin-1-mediated regulatory circuits established during acute *T. cruzi* infection (43) were unlikely to explain improved control of parasite growth in *L. donovani*-infected *Lgals1^−/−^* mice. Of note, despite engrafting mice with a 90:10 mix of WT and *Lgals1^−/−^* BM, respectively, we consistently found that *Lgals1^−/−^* leukocytes comprised 20–25% of the immune cell compartment (Figure 8A), although the ratio of CD4^+^ T cells in WT and *Lgals1^−/−^* compartments remained the same (approximately 30%). Together, these results indicate the enhanced CD4^+^ T cell IFNγ response observed in the liver of *Lgals1^−/−^* animals involved direct galectin-1 signaling to CD4^+^ T cells. Furthermore, the lack of effect on hepatic CD4^+^ T cell IFNγ production in mice with galectin-1-deficient DC (i.e., DT-treated CD11c.DOG:*Lgals1^−/−^* chimeras), compared to controls (Figure 7C), indicated that direct galectin-1 signaling by DCs was not responsible for the increased hepatic CD4^+^ T cell IFNγ production observed in *Lgals1^−/−^* mice (Figure 5C).

## Discussion

A substantial amount of work on the immunoregulatory activity of galectin-1, and indeed the other members of the galectin family, has been conducted in the context of cancer and associated illnesses (20). As a result, the anti-inflammatory properties of galectin-1 have been well described (28, 29, 52). Thus, the primary focus of this study was to outline the contribution of galectin-1 signaling during experimental VL, with specific focus on the involvement of galectin-1 in CD4^+^ T cell IFNγ and IL-10 production. Our studies centered on CD4^+^ T cells because we and others have previously identified CD4^+^ T cells, but not CD8^+^ T cells, to be critical for control of *L. donovani* growth during primary infection (47, 53). CD8^+^ T cells are important for protection against re-infection the liver (53), and protection in some vaccination strategies (54, 55). Here, we report that galectin-1 plays a limited role in promoting IL-10 production by CD4^+^ T cells during *L. donovani* infection. However, we found galectin-1-mediated suppression of effector cytokine production by hepatic Th1 cells following infection. This finding was at odds with the proposed immunoregulatory network involving galectin-1 outlined by others both *in vitro* (29), and more recently *in vivo* (43). The latter study reported that *Lgals1^−/−^* mice had reduced mortality and parasite burden following *T. cruzi* infection, compared to WT controls. However, protection was associated with disruption of an immunoregulatory mechanism involving DC-mediated expansion of Treg cells (43). We found the impact of galectin-1 signaling on Foxp3^+^ Treg cell IL-10 production was minimal in experimental VL. Given galectin-1’s role in promoting Treg cell suppressive functions (43), our results indicate either a different role for galectin-1 in hepatic parasite clearance during *L. donovani* infection or an IL-10-independent mechanism of galectin-1-mediated Treg cell modulation. Our data from BM chimeric mice showed increased frequencies of Treg cells when either DCs or Treg cells lacked galectin-1 expression. This finding is at odds with what has been reported during acute *T. cruzi* infection (43), where DC galectin-1 expression promoted Treg cell function, and suggests the distinct roles for galectin-1 in different inflammatory settings. In addition, Treg cells have previously been shown to use multiple mechanisms of immune suppression, including TGFβ, IL-35, and CTLA4 (56, 57), and the impact of galectin-1 deficiency on these features of Treg cell functions during experimental VL still needs to be addressed. Another unexplored galectin-1-mediated mechanism of immune regulation in our study was the induction of apoptosis. Again, this needs examination, given the initial reports on the role of galectin-1 in T cell apoptosis (25). Similarly, we have previously reported that γδ T cell-derived IL-17A suppressed early hepatic anti-parasitic immunity following *L. donovani* infection (10, 47, 49), and although we have never observed IL-10 production by these cells, we cannot exclude a role for galectin-1 in this response.

Despite the limited impact of galectin-1 deficiency on Tr1 cell development during *L. donovani* infection, Th1 cell numbers in the liver were increased in *Lgals1^−/−^* mice 14 days after *L. donovani* infection, and this correlated with decreased parasite load during acute hepatic infection. Therefore, the action of galectin-1 may depend on the extent of antigen availability and consequent T cell activation status, rather than the induction of IL-10 during experimental VL. Furthermore, changes in galectin-1 expression by various immune cells were most prominent in the liver during the first 14 days of infection, which may have contributed to the tissue-specific impact on parasite control we observed. Our findings with mixed BM chimeras indicate that galectin-1 influences IFNγ production by CD4^+^ T cells during the acute phase of hepatic *L. donovani* infection in a cell intrinsic manner. Previous studies (30, 43) suggest that DCs mediate immunoregulatory effects of galectin-1. This notion was supported by improved parasite clearance early during infection in the liver in *Lgals1^−/−^* animals, at a time when effector CD4^+^ T cell responses were being initiated. However, depletion of DC in the presence of either WT or galectin-1-deficient cells did not influence effector T cell function following *L. donovani* infection. Therefore, our data indicate that DC-expressed galectin-1 is unlikely to contribute to the augmented Th1 responses observed in the liver of *Lgals1^−/−^* animals. However, we cannot exclude the possibility that galectin-1 expressed by other potential antigen-presenting cells, such as macrophages and/or monocytes, might influence T cells responses during experimental VL, an idea supported by the high levels of galectin-1 expression by these cells in the liver following infection. In addition, distinct requirements for DC activity in specific tissue sites by discrete DC subsets in cancer models and *T. cruzi* infection may also help explain lack of requirement for DC-dependent galectin-1-mediated immune regulation in experimental VL.

It is not clear why galectin-1 is expressed on multiple immune cell populations in the liver during *L. donovani* infection, and only influences parasite control in this tissue at the acute stage of infection. One potential explanation is that galectin-1 is preferentially expressed by migrating CD4^+^ T cell populations that would be expected to be more prominent in the liver during experimental VL. Alternatively, the extent of immune dysregulation in the chronically infected spleen means that abrogation of galectin-1 activity is not, by itself, enough to overcome the *status quo* of impaired cellular responses in this organ. Conversely, the liver, with its delicate balance of pro- and anti-inflammatory responses (4, 50, 51), could be susceptible to more subtle alterations in the immunoregulatory environment. One caveat that must be highlighted, however, is that the flow cytometric analysis of galectin-1 expression in our study does not discern between dimeric and monomeric galectin-1, with obvious implications for interpretation of function. Thus, the mechanism by which galectin-1 impairs parasite clearance remains unclear. Our data indicate a CD4^+^ T cell intrinsic role for galectin-1 in suppressing IFNγ production. As mentioned previously, monomeric galectin-1 can modulate cell functions by interacting with Ras family proteins (22), and given that suppression of Ras protein activity by farnesyltransferase inhibitors caused reduced IFNγ production by mouse and human CD4^+^ T cells (58), the manipulation of Ras protein by galectin-1 in CD4^+^ T cells is one possible mechanism for inhibition of IFNγ production in *L. donovani*-infected mice. An important finding in our studies was that *Lgals1^−/−^* mice exhibit improved parasite clearance prior to the establishment of strong effector T cell activity (day 7 p.i.), thus indicating a broader immunoregulatory potential of galectin-1, and the extent of which appears to depend on the infected tissue. Both the precise mechanism of galectin-1-mediated suppression of IFNγ production by CD4^+^ T cells and the broader roles of galectin-1 on other immune cells require further study. One approach could be to block CD45R, as this has previously been shown to be required for galectin-1-mediated functions (25), and might provide novel insights to the mechanism of action of galectin-1 in experimental VL.

In summary, we demonstrate that galectin-1 is an important immunoregulatory molecule in experimental VL. Unexpectedly, galectin-1 did not appear to play a major role in Tr1 cell development or function. The main effect of galectin-1 on anti-parasitic immunity appeared to the suppression of Th1 cells. However, the relatively early effects of galectin-1-deficiency during infection also suggest effects of galectin-1 on innate cell activity, although this is unlikely to be on DCs. Together, these findings provide new insights into the role of galectin-1 in modulating immunity during infectious diseases and highlight both tissue- and disease-specific roles for this molecule.

## Ethics Statement

All animal procedures were conducted with the approval of the QIMR Animal Ethics Committee under the animal ethics number A02-634M and in accordance with the “Australian Code of Practice for the Care and Use of Animals for Scientific Purposes” (Australian NHMRC, Canberra).

## Author Contributions

PB, MO, and CRE designed, performed, and analyzed the work, and wrote the paper. FR, RK, CLE, RF, SN, MS, YW, FA, and AH performed the work and analyzed data.

## Conflict of Interest Statement

The authors declare that this research was conducted in the absence of any commercial or financial relationships that could be construed as a potential conflict of interest.
